# Germ soak water as nutrient source to improve fermentation of corn grits from modified corn dry grind process

**DOI:** 10.1186/s40643-017-0170-8

**Published:** 2017-08-23

**Authors:** Ankita Juneja, Deepak Kumar, Vijay Singh

**Affiliations:** Department of Agricultural and Biological Engineering, University of Illinois at Urbana-Champaign, Urbana, IL 61801 USA

**Keywords:** Ethanol, Dry grind, Granular starch hydrolysis, Yeast nutrition, Fermentation

## Abstract

Corn fractionation in modified dry grind processes results in low fermentation efficiency of corn grits because of nutrient deficiency. This study investigated the use of nutrient-rich water from germ soaking to improve grits fermentation in the conventional dry grind and granular starch hydrolysis (GSH) processes. Comparison of germ soak water with the use of protease and external B-vitamin addition in improving grits fermentation was conducted. Use of water from optimum soaking conditions (12 h at 30 °C) resulted in complete fermentation with 29 and 8% higher final ethanol yields compared to that of control in conventional and GSH process, respectively. Fermentation rate (4–24 h) of corn grits with germ soak water (0.492 v/v-h) was more than double than that of control (0.208 v/v-h) in case of conventional dry grind process. The soaking process also increased the oil concentration in the germ by about 36%, which would enhance its economic value.

## Background

Bioethanol is considered as one of the most promising renewable alternatives to petroleum-based transportation fuel. In the conventional dry grind process, corn starch is liquefied to dextrins at high temperature and pressure, which are further converted to glucose during the saccharification process. Glucose is simultaneously fermented to ethanol by yeast, and this combined process is known as simultaneous saccharification and fermentation (SSF). In an alternate approach, granular starch hydrolyzing enzymes (GSHE) can directly hydrolyze the raw granular starch into glucose at low temperatures, without the need of liquefaction step. At the end of both processes, remaining non-fermentable components (germ, fiber, protein, and residual starch) are recovered as DDGS (distillers dried grains with solubles), a coproduct primarily used as ruminant animal food. Fractionation of corn to recover germ and pericarp, prior to hydrolysis, is one way to generate valued coproducts and simultaneously improve nutritional value of DDGS (low fiber due to removal of pericarp) (Murthy et al. 2006a, b). Germ and pericarp obtained from the modified process can be refined to obtain valuable products including corn oil from corn germ and corn fiber oil from pericarp fiber. Corn fiber oil has very high economic value because its constituents have nutraceutical properties (Moreau et al. 1996; Murthy et al. 2006b). Grits obtained after germ and pericarp removal contain relatively high amount of starch, which would produce higher ethanol concentrations compared to whole corn at same solid loadings.

However, removal of germ during corn fractionation also removes the soluble proteins and micronutrients present in the germ that are essential for yeast during the fermentation process. Also the lipids, present in germ and the aleurone layer below the pericarp, are essential to maintain membrane integrity and yeast activity, especially during high glucose and ethanol concentrations. Murthy et al. (2006a) reported that both initial rate of fermentation and final ethanol concentrations were low for endosperm obtained from 3D process compared to those from wet fractionation (E-milling). One way to address this problem to some extent is addition of protease enzymes. Addition of proteases causes hydrolysis of the protein matrix surrounding the starch granules, which produces free amino nitrogen (FAN) as well as improve accessibility of starch to enzymes. Fermentation efficiency can also be improved by adding external nutrition, such as yeast extract, lipid supplementation, and B-vitamin complex. However, both protease enzymes and external nutrient add up to the cost of the process and counter the benefits of fractionation. One potential cost-effective approach could be the extraction of these nutrients from the recovered germ, as suggested by Murthy et al. (2006a). The study reported that the water obtained after soaking of fractionated germ (2 h soaking) resulted in increase of final ethanol concentrations from 12.3 to 14.7% (v/v) during conventional dry grind processing of corn grits.

This study aims to investigate this approach in detail and optimize the process conditions (germ soaking time and amount) to maximize the fermentation rate and final ethanol concentrations of corn grits during conventional dry grind as well as GSH process. Germ water was obtained from two soaking conditions and its effect on fermentation performance of corn grits was compared to those from control, protease addition, and B-vitamin addition. Combination of germ water and B-vitamins was also investigated to determine the maximum achievable ethanol efficiency. Composition of raw germ and germ after soaking was also evaluated to determine the changes in oil concentrations.

## Methods

### Materials

Flaking grits and germ samples were obtained from a commercial corn dry-milling plant (Bunge, Danville, IL, USA). Samples were stored in refrigerator at 4 °C till analysis. All enzymes including conventional α-amylase (Spezyme RSL with reported activity of 20,100 NLC/g), conventional glucoamylase [distillase SSF, with reported activity of 380 GAU/g (GAU: glucoamylase unit)], GSHE (Stargen 002), and Protease (Fermgen) are commonly used commercial enzymes and were generously donated by DuPont Industrial Biosciences (Palo Alto). GSHE contained α-amylase from *A. kawachi* expressed in *T. reesei* and glucoamylase from T. *reesei*, and had an activity of >570 GAU/g. Protease enzyme contained fungal protease obtained from genetically modified selected strain of *T. reesei,* with an activity of >1000 SAPU/g (SAPU is spectrophotometric acid protease units). Conventional active dry yeast (ethanol red) was obtained from the Fermentis-Lesaffre Yeast Corporation (Milwaukee, Wisconsin).

### Corn grits and germ composition

Composition analysis of corn grits, raw germ, and soaked germ was performed as per American Association of Cereal Chemists International (AACCI) standard procedures. The moisture content of corn grits was determined by drying the samples in hot air oven at 135 °C for 2 h (AACC international approved method 44-19.01). Corn grits and germ (before and after soaking) were analyzed for crude protein content (method 990.03), oil (method 920.39), and neutral detergent fiber (method 2002.04) in a commercial analytical laboratory (Illinois crop improvement association, Champaign, IL, USA). All analyses were conducted in duplicates. Starch content in the ground corn grits was determined using acid hydrolysis method (Vidal et al. 2009). Briefly, about 1 g of ground corn samples (~1 g) were mixed with 50 mL dilute HCl (0.4 N) in 100 mL autoclavable bottles, and the slurry was autoclaved at 126 °C for 1 h (Napco Model 9000D, Thermo Fisher 157 Scientific, Waltham, MA). Pure glucose and starch samples were used to determine glucose recovery factors. After cooling, 1 mL aliquot samples was withdrawn and centrifuged at 1500×*g* for 5 min (Model 5415 D, Brinkmann–Eppendorf, Hamburg, Germany). The supernatants were analyzed in the HPLC for glucose determination.

### Dry grind process

**Fig. 1:**
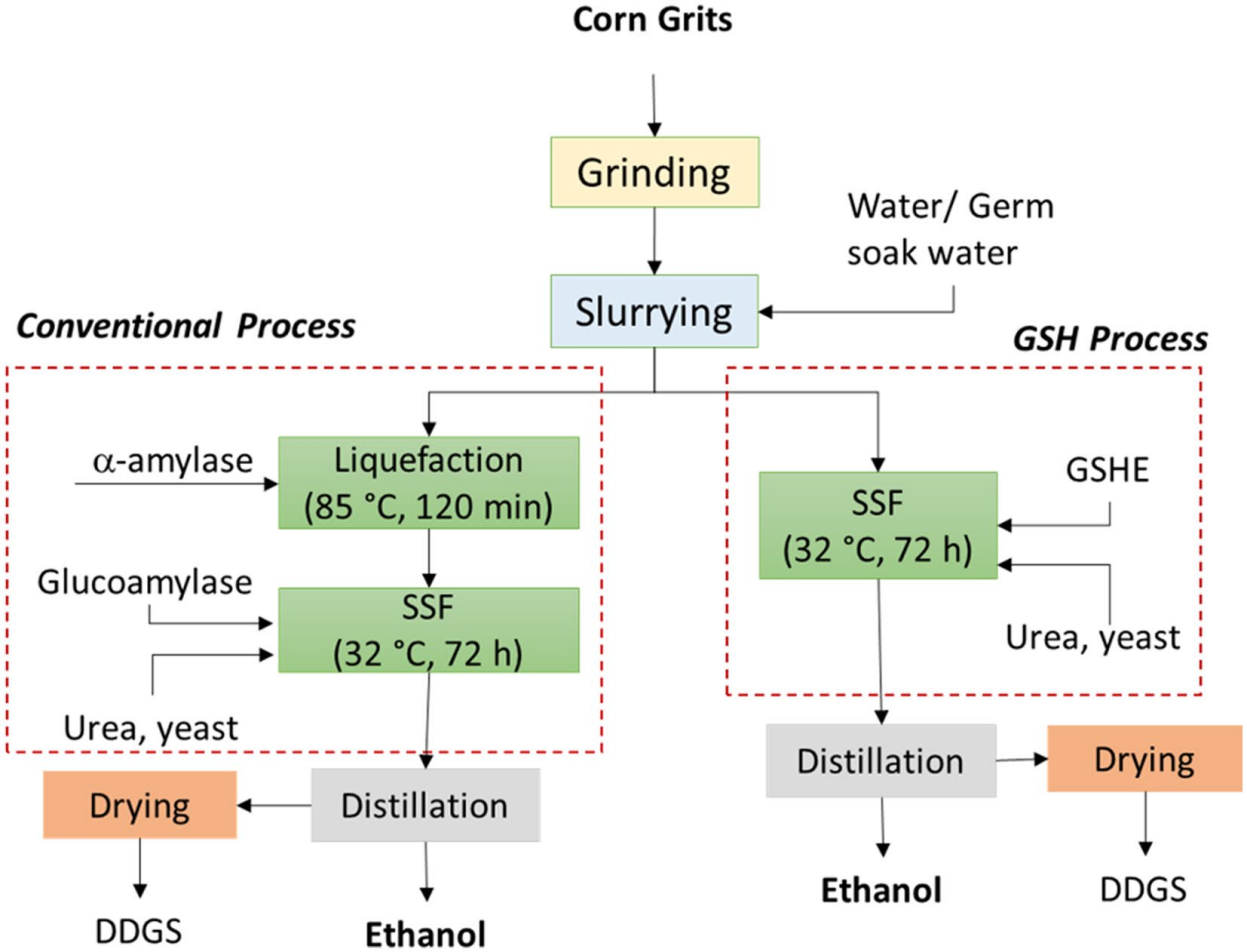
Schematic of laboratory-scale conventional dry grind and GSHE process for ethanol production

A simple schematic of lab-scale dry grind and GSH process is shown in Fig. 1. Corn grits were ground in a laboratory-scale mill (model MHM4, Glen Mills, Clifton, NJ) at 500 rpm and using a 0.5-mm screen. All experiments were performed at 250 mL scale in 500 mL stainless steel reactors in duplicate. Ground grits were mixed with water or germ-soaked water (Table 1) to make a slurry with 25% solids on dry basis. For liquefaction, the pH of the slurry was adjusted to 5.1 using 10 N sulfuric acid and 16 µL α-amylase was added, as per manufacturer’s recommendations. The liquefaction was performed in Labomat Incubator (Labomat BFA-12, Werner Mathis AG, Switzerland) at 85 °C for 120 min with continuous shaking. Heating and cooling of the samples in the incubator were at the rate of 3 °C/min (this time was additional to 90 min of liquefaction time). The liquefied slurry was then prepared for simultaneous saccharification and fermentation (SSF). The pH of the slurry was adjusted to 4.3 using 10 N sulfuric acid and GA (54.7 mL), urea (0.4 mL of 50% w/v solution), and yeast inoculum (2 mL) were added. Yeast inoculum was prepared by dispersing 5 g of active dry yeast in 25 mL of distilled water and agitated at 100 rpm and 32 °C for 20 min. The broth was fermented at 32 °C for 72 h in an automatic incubator with continuous agitation (150 rpm). Samples (2 mL) were drawn at 4, 8, 24, 48, and 72 h to monitor the fermentation.

**Table 1:** Description of treatments investigated in processing corn grits using conventional dry grind and GSH process

Treatment	Description	DI water (% of liquid in slurry)	Germ soak water (% of liquid in slurry)	Germ soaking conditions		Protease	B-vitamin
				Temp (°C)	Time (h)		
T1	Control	100	0	–	–	No	No
T2	Control with protease	100	0	–	–	Yes	No
T3	Partial germ water	66.66	33.33	30	2	No	No
T4	Partial germ water—long time	66.66	33.33	30	12	No	No
T5	Full germ water	0	100	30	12	No	No
T6	B-vitamin	100	0	–	–	No	Yes
T7	Partial germ water and B-vitamin	66.66	33.33	30	12	No	Yes

Germ soak water was obtained by soaking the germ under two conditions: (i) 30 °C for 2 h and (ii) 30 °C for 12 h (Table 1). In each case, 25 g of germ was mixed in 250 mL of deionized (DI) water in 500 mL flasks and was incubated as per conditions mentioned in Table 1, with continuous shaking at 125 rpm. After soaking, the liquid was vacuum-filtered through Whatman No. 4 filter paper and used to make slurry as described in Table 1. Two dosages of germ water were investigated: (1) one-third (33.33%) of total liquid in slurry, referred as partial germ water (treatments T3, T4, T7 in Table 1), (2) 100% of liquid in slurry, referred as full germ water (treatment T5 in Table 1) in the article. The first case (partial germ water) represents the water obtained from the soaking of germ proportional (10%) to corn grits used in the experiment. In the current study, 62.5 mL of germ water was added in total 250 mL slurry (62.5 g corn grits and 187.5 mL liquid). Full germ water case was investigated to determine the effect of adding excess nutrients on the fermentation efficiency. Other than germ soak water, two additional set of treatments (T6 and T7) were performed by addition of B-vitamins. In treatment T6, conditions were similar to that of control, except excess of vitamin B12 and B-complex were added at the start of SSF process. In the case of treatment T7, combined effect of germ soak water and B-vitamins was investigated and excess of vitamin B12 and B-complex was added in addition to germ soak water (Table 1).

### GSH process

The front-end operations (cleaning, grinding, and slurry making) were similar to that of conventional dry grind process described above (Fig. 1). Liquefaction step is not required in this process. The slurry prepared was adjusted to a pH of 4.1 using 10 N sulfuric acid, and GSHE (0.234 mL), urea (0.4 μL of 50% w/v solution) and yeast inoculum (2 mL) were added. Yeast inoculum was prepared as described in the previous section. The slurry was incubated at 32 °C for 72 h in an automatic incubator with continuous agitation (150 rpm), and 2 mL of samples were drawn at 4, 8, 12, 24, 48, and 72 h to monitor the fermentation. This process was also investigated for all conditions presented in Table 1.

### HPLC analysis

Samples collected were centrifuged at 9729 g (5415 D, Brinkmann Eppendorf, Hamburg, Germany) for 10 min, and clear liquid was passed through 0.2 µm syringe filters (nylon Acrodisc WAT200834, Pall Life Sciences, Port Washington, NY) into 150 µL HPLC vials. The vials were immediately stored at −20 °C until analysis. The filtrate was then analyzed using HPLC with an ion-exclusion column (Aminex HPX-87H, Bio-Rad, Hercules, CA, USA). The mobile phase was 0.005 M sulfuric acid at 50 °C at a flow rate of 0.6 mL/min. The amounts of sugars, alcohols, and organic acids were quantified using a refractive index detector and multiple standards.

### Fermentation rate and ethanol yield

For each treatment, ethanol and glucose concentration was measured at every time point as described above and were plotted against time. Fermentation rates (ethanol production rates) between different time points were calculated using Eq. 1.

1 $${\text{Fermentation rate}} = \frac{{E_{{t_{2} }} - E_{{t_{1} }} }}{{t_{2} - t_{1} }},$$

where $E_{{t_{2} }}$ and $E_{{t_{1} }}$ are ethanol concentrations (% v/v) at fermentation times *t*_2_ and *t*_1_, respectively.

Starch-to-ethanol conversion efficiencies were calculated as the ratio of actual ethanol yields with the theoretical ethanol yield (Eq. 2).

2 $$\eta_{\text{EtOH}} = \frac{{E_{\text{EtOH}} }}{{E_{{{\text{Th\_EtOH}}}} }}* 100,$$

where *E*_Th_EtOH_ is theoretical ethanol yield, L/kg dry corn grits; *E*_EtOH_ is the actual ethanol yield, L/kg dry corn grits.

Theoretical yields were estimated based on the starch content, assuming complete starch conversion and 100% fermentation efficiency. Actual ethanol yields were determined by calculating liquid volume in final slurry at end of fermentation (Kumar and Singh 2016). Final slurry was weighed and a sample of the slurry was dried in hot air oven at 105 °C till constant weight was achieved (~24 h) to estimate the solid percent in the slurry. The actual ethanol yields were calculated using following Eqs. 3–5.

3 $$W_{\text{L}} = W_{\text{slurry}} *(1 - S_{\text{slurry}} )$$

4 $$V_{\text{EtOH}} = \frac{{W_{\text{L}} }}{{\rho_{{{\text{H}}_{ 2} {\text{O/EtOH}}}} }}*C_{\text{EtOH}}$$

5 $$E_{\text{EtOH}} = \frac{{V_{\text{EtOH}} }}{{W_{C} *\left( {1 - {\text{MC}}_{\text{C}} /100} \right)}},$$

where *W*_L_ is the weight of liquid in the fermented slurry, g; *W*_slurry_ is the weight of fermented slurry, g; *S*_slurry_ is the solid fraction in the slurry; *V*_EtOH_ is the volume of ethanol produced, mL; $\rho_{{{\text{H}}_{ 2} {\text{O/EtOH}}}}$ is the density of water–ethanol mixture (g/L) at final ethanol concentration; *C*_EtOH_ is the final ethanol concentration, mL/L; *E*_EtOH_ is the actual ethanol yield, L/kg; MC_C_, is the moisture content in grits, %; and *W*_L_ is the weight of the grits.

### Statistical analysis

Analysis of variance (1-way ANOVA) and Fisher’s least significant difference (LSD) tests were used to compare the glucose and ethanol concentrations (SAS version 9.3). The level selected to show the statistical significance in all cases was 5% (*P* < 0.05).

## Results and discussion

### Composition of corn grits

Starch content in the corn grits was estimated as 86.5% on dry basis. Crude protein, oil, and neutral detergent fiber (NDF) were 6.1, 0.6, and 0.9% (dry basis), respectively. Based on this composition, the theoretical ethanol yield was calculated 0.62 L/kg dry corn grits (4.17 gal/bu).

### Conventional dry grind process

#### Effect of germ soak water

**Fig. 2:**
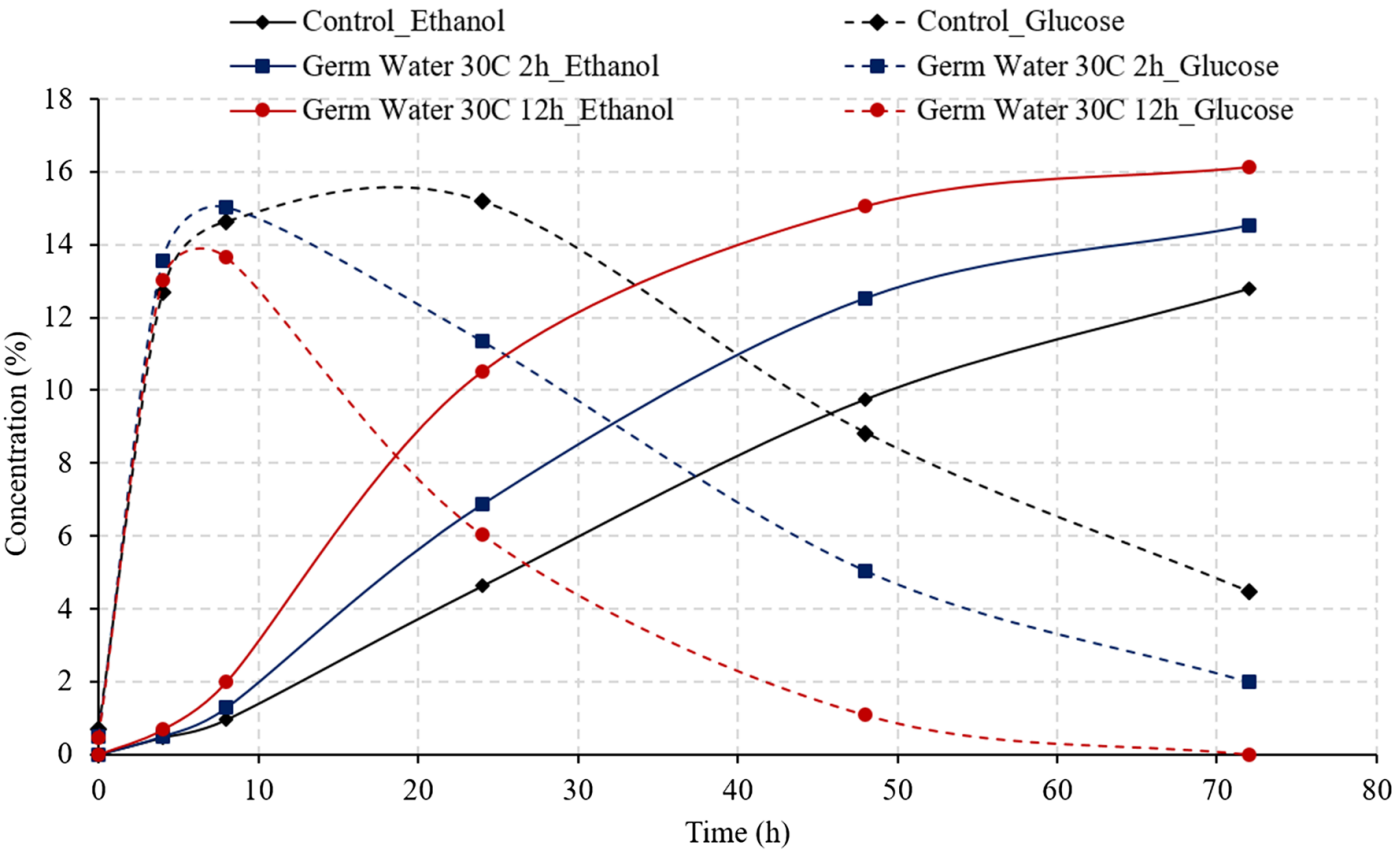
Effect of germ water addition on fermentation of corn grits in conventional dry grind process. (Ethanol concentrations in % v/v and glucose concentration % w/v)

Figure 2 illustrates the comparison of ethanol and glucose concentration during SSF of corn grits, for control and germ water addition from two soaking conditions. As expected, addition of germ water improved the fermentation profile compared to that of control. The addition of germ water from soaking at 30 °C for 2 h resulted in an increase in the final ethanol concentration from 12.79 to 14.52% (v/v), which was similar to as observed by Murthy et al. (2006a). Likewise, there was unconverted glucose (2% w/v) observed at the end of the fermentation. However, the addition of germ water from longer soaking conditions (30 °C for 12 h) resulted in complete fermentation with a final ethanol concentration of 16.14% and no residual glucose. The average final ethanol concentration of the 12-h germ-soaked water was 28.3 and 12.9% higher than the control and 2-h germ-soaked water, respectively. Final glycerol production in case of germ water (30 °C for 12 h)-supplemented samples was about 23% less than that of control (1.16 vs. 1.51%).

About 4.5% glucose remained unconverted in the case of control, which along with high glycerol production resulted in very low starch-to-ethanol conversion efficiency (62.4%). Efficient fermentation with germ water (30 °C and 12 h) addition led to an increase in conversion efficiency to 82.7%, which was 12% (in relative terms) higher than that in the case of addition of 2-h germ-soaked water (73.8%). Since the glucose released in the first 8 h is similar for all three conditions (Fig. 2), it can be stated that the increased rate of fermentation in the 12-h germ-soaked water is due to the better functioning of the yeast in the presence of micronutrients and free amino acids present in germ soak water. These results indicate that longer soaking resulted in leaching out more nutrients that improved the yeast performance and led to high ethanol yields and fermentation rates. Due to the lack of these micronutrients in the control, fermentation was observed to be slowest among all treatments. Ethanol yields from control, treatment with 2-h germ water and treatment with 12-h germ water were estimated as 0.39, 0.46, and 0.51 L/kg dry grits (2.6, 3.1, and 3.5 gal/bu) respectively.

#### Germ water vs. protease addition

**Fig. 3:**
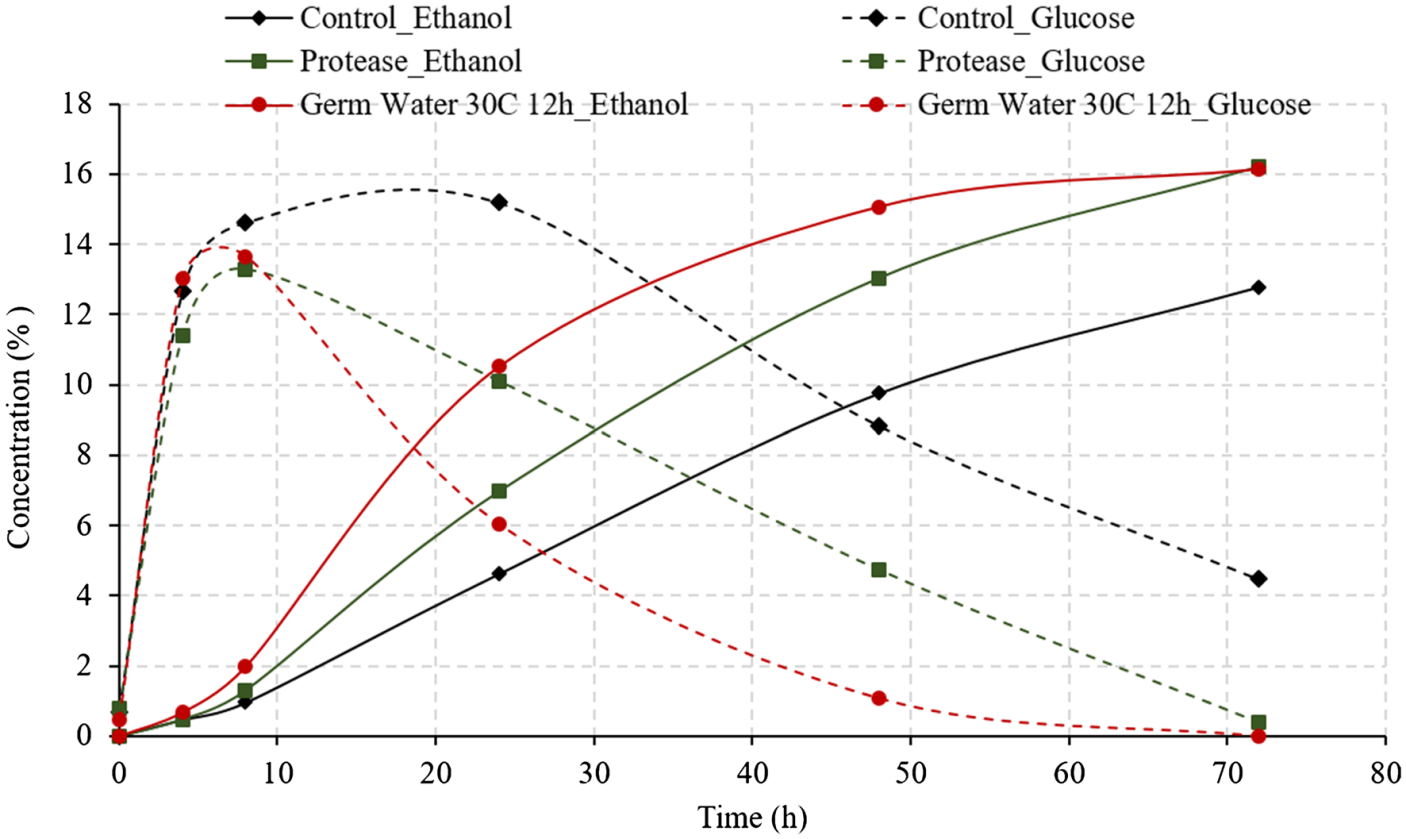
Comparison of germ soak water and protease enzyme on fermentation profile of corn grits during conventional dry grind process. (Ethanol concentrations in % v/v and glucose concentrations % w/v)

Earlier studies have shown the addition of protease enzymes increases the fermentation rate and ethanol yield in the dry grind process (Johnston and McAloon 2014; Vidal et al. 2009). However, protease are relatively expensive enzymes and add up significant cost in the process (Wang 2008). Figure 3 illustrates the fermentation profile (glucose and ethanol concentrations) of control, treatment with protease addition, and germ soak water (30 °C, 12 h) addition (treatment T1, T2, and T4 in Table 1) during SSF of corn grits. As expected, the addition of protease enzymes improved the fermentation efficiency compared to that of control and resulted in final ethanol concentration 16.2% (v/v) compared to only 12.79% for control. As discussed in earlier section, germ water from new soaking conditions (30 °C, 12 h) resulted in complete fermentation with 16.14% ethanol (same as that of protease); however, the initial fermentation rates in case of germ soak water were observed to be higher (0.49 vs. 0.32 v/v-h in 4–24 h). Ethanol yields of 0.51 and 0.52 L/kg dry grits (3.45 and 3.46 gal/bu) were similar for treatments with germ water supplementation and protease addition. The results indicated that the addition of 12-h germ-soaked water could potentially replace the protease enzyme, with even higher ethanol production rate.

#### Effect of water amount

To further investigate the process, the amount of germ water addition was also varied. Instead of adding one-third of total liquid during slurry formation, the slurry was prepared with 100% germ soak water (30 °C, 12 h) in this case. Ethanol and glucose concentrations during SSF of corn grits without and with addition of two dosages of germ soak water (partial and full germ soak water as explained in Table 1) are given in Table 2. Final ethanol concentration was observed similar for both treatments (partial germ water and full 100% germ water). However, the ethanol production rate in case of 100% germ soak water was higher (0.54 vs. 0.49% v/v/h) than of partial germ water case. No residual sugars in the broth indicated that maximum ethanol potential had been reached. Ethanol yields of 0.51 L/kg dry biomass were similar in both treatments. Considering the similar final ethanol concentrations and yields, it can be interpreted that micronutrients in partial germ water slurry were sufficient for the yeast and there would not be a huge advantage of making slurry with only germ soak water.

**Table 2:** Effect of using partial vs. full germ water on the glucose and ethanol concentrations of corn grits during conventional dry grind process

	Treatment	Time (h)					
		0	4	8	24	48	72
Ethanol (% v/v)	Control	0.00	0.48	1.00	4.72	9.61	12.58
	Partial GW	0.00	0.68	1.99	10.52	15.07	16.14
	Full GW	0.00	0.69	2.29	11.41	16.03	16.32
Glucose (% w/v)	Control	0.64	13.84	15.45	15.37	9.17	4.88
	Partial GW	0.47	13.01	13.64	6.04	1.08	0.00
	Full GW	0.67	7.02	8.79	1.77	0.10	0.00

#### Effect of B-vitamins

**Fig. 4:**
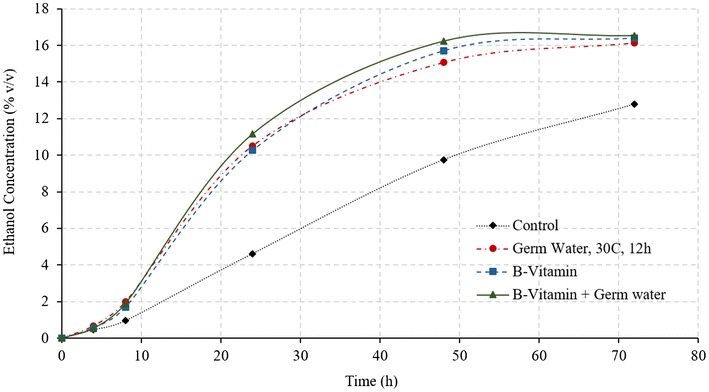
Ethanol concentrations during fermentation of corn grits in conventional dry grind process with various treatments

**Fig. 5:**
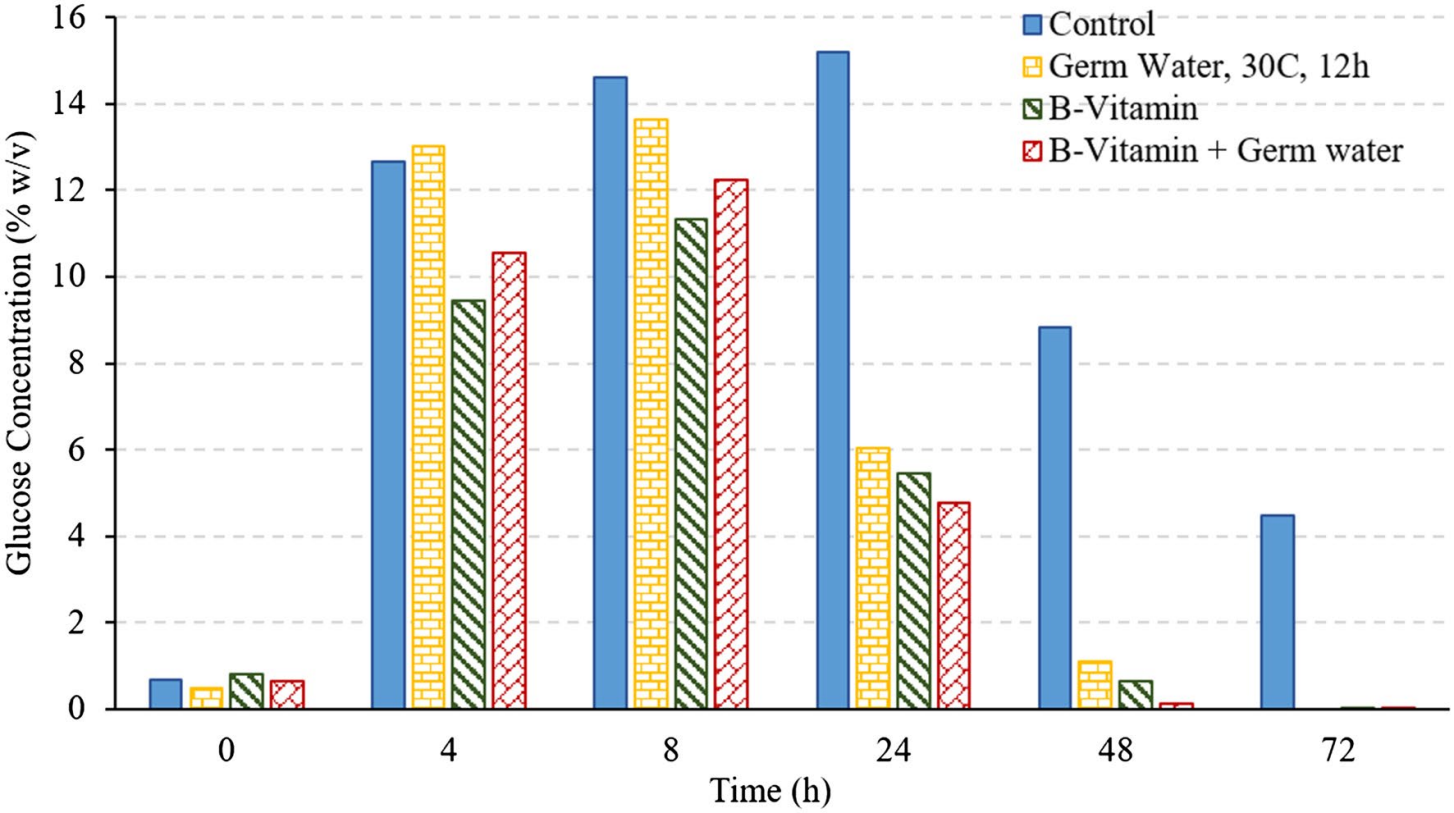
Glucose concentrations during fermentation of corn grits in conventional dry grind process with various treatments

Vitamins are essential for yeast metabolism and functioning, however, yeast cannot synthesize many of these vitamins. B-vitamin complex consists of essential coenzymes involved in carbohydrate metabolism and provides necessary metabolic precursors for yeast growth. Other vitamins such as nicotinic acid and pantothenic acid are also helpful in improving yeast performance (White 2012). Riboflavin (vitamin B2) is essential for lipid synthesis, and vitamin B6 is essential for nitrogen metabolism in yeast (Murthy et al. 2006a). Supplementation of vitamin B1, B12, and B-complex have previously shown to increase the ethanol concentrations during fermentation (Laser 1941; Murthy et al. 2006a). Effect of addition of B-vitamins on the fermentation profile of the corn grits was investigated under two conditions: (i) control with addition of excess of B12 and B-complex vitamins, and (ii) addition of both germ soak water (30 °C, 12 h) and excess B-vitamins (treatments T6 and T7 in Table 1). These conditions would answer two questions: (i) can germ water addition improve fermentation performance similar to that of B-vitamins, and (ii) what is the maximum achievable fermentation improvement. Results from these conditions are illustrated in Figs. 4 and 5.

Final ethanol concentrations and yields with the use of only germ soak water were similar to those of treatments with addition of B-vitamins and both germ water and B-vitamins (*P* > 0.05) (Fig. 4; Table 3). These results indicate that germ soak water has sufficient nutrients required to achieve similar ethanol profiles as with B-vitamins. The initial ethanol production rate was higher in the treatment using both germ water and B-vitamins. In all the three cases (germ water, B-vitamin, and germ water plus B-vitamin), the glucose concentration at the end of the fermentation is negligible, which indicates complete fermentation at 72 h. These results suggest that addition of germ water can replace the need for the addition of expensive vitamins, and maximum fermentation rate can be achieved by adding both germ water and B-vitamins.

**Table 3:** Ethanol yields and conversion efficiencies for all treatments in dry grind process

Treatments	Final ethanol concentration (%)	Ethanol conversion efficiency (%)	Fermentation rates (% v/v/h)
Control	12.79 d	62.44 e	0.208 d
Protease	16.20 a b	82.83 a b c	0.324 c
Germ soak water 2 h 30 °C	14.52 c	74.10 d	0.318 c
Germ soak water 12 h 30 °C-PS	16.14 a b	82.67 a b c	0.492 b
Germ soak water 12 h 30 °C-FS	16.32 a	82.14 b c	0.536 a
B-vitamins	16.39 a	84.02 a	0.484 b
B-vitamins + Germ soak water^a^	16.54 a	83.43 ab	0.531 a

Means followed by the same letter in one column are statistically not different (at *P* < 0.05)

*PS* partial slurry, *FS* full slurry

^a^Partial slurry of 12 h 30 °C germ soak water

Final ethanol concentrations, fermentation rates (4–24 h), and ethanol conversion efficiencies for all treatment have been compiled in Table 3. Except for control and germ water from soaking at 30 °C and 2 h, the ethanol conversion efficiency was more than 80% in all treatments. Although conversion efficiency is similar in all other cases, the fermentation rate (4–24 h) was maximum for full germ slurry and treatment using both germ water and B-vitamins.

### Granular starch hydrolysis process

Considering the advantages (low energy use and low glucose inhibition) and increasing trend of granular starch hydrolysis process in corn ethanol industry, it was important to investigate the effect of germ soak water on yeast performance in GSH process also. Performance of germ water for all conditions listed in Table 1 was studied and compared with control, protease addition, and B-vitamin addition.

#### Effect of germ soak water

**Fig. 6:**
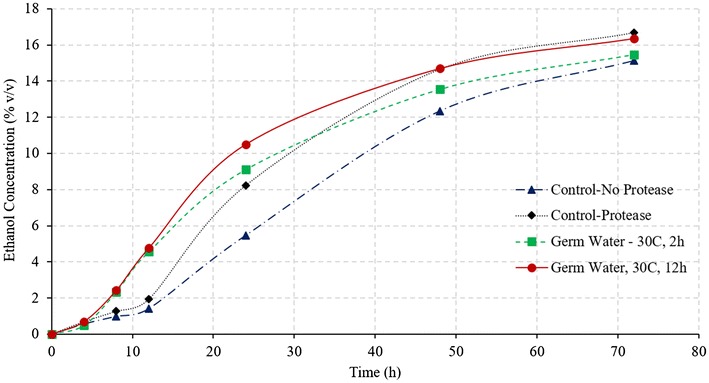
Comparison of ethanol concentrations among control, treatment with protease addition, and treatment with germ water addition during fermentation of corn grits in GSH process

**Fig. 7:**
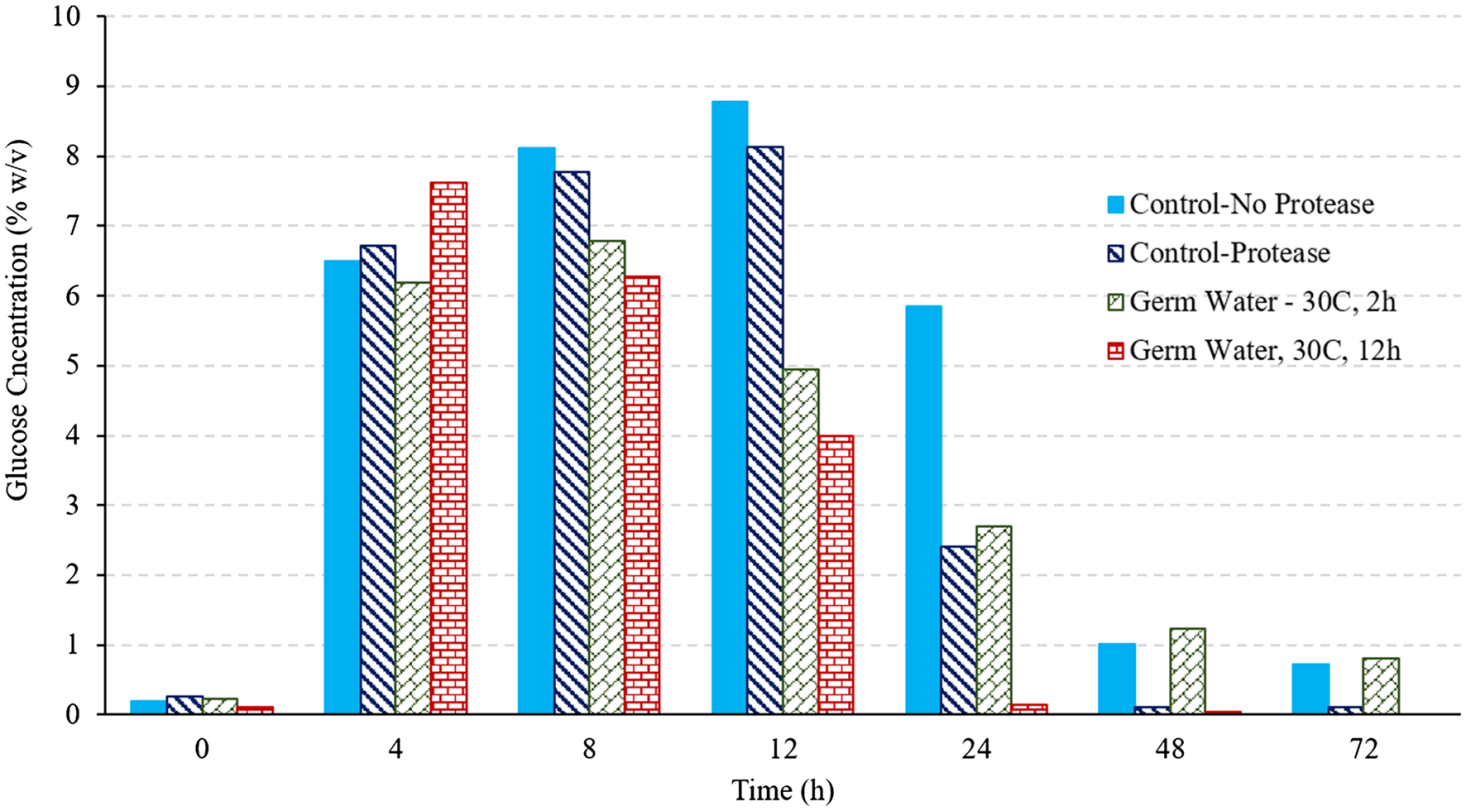
Comparison of glucose concentrations among control, treatment with protease addition, and treatment with germ water addition during fermentation of corn grits in GSH process

Similar to conventional dry grind process, germ water addition improved the fermentation performance compared to that of control (Figs. 6, 7). The improvement was better with addition of germ water obtained from soaking at 30 °C for 12 h. With addition of germ water from soaking at 30 °C for 2 h, the ethanol production rate was higher (0.43 vs. 0.25% v/v-h), however, the final ethanol concentration was similar as that of control (Table 4). The average final ethanol concentration in treatment supplemented with germ water obtained from longer soaking (12 h) was 8.3% higher than the control (16.35 vs. 15.10% v/v), with negligible unconverted glucose at the end of fermentation. About 0.72% (w/v) and 0.82% (w/v) glucose remained unconverted in cases of control and germ water from soaking for 2 h (Fig. 7). The increase in final ethanol concentration in this process (8.3%) was less than that observed in case of conventional process (28.3%). This was attributed to the higher ethanol concentrations obtained with GSH enzymes in control due to relatively low glucose inhibition. The peak glucose concentration for the conventional process was almost double (16.12%) compared to that from GSH process (8.77%). The conversion was higher because of different enzymes loadings and synergistic action α-amylase and glucoamylase in the GSH process. Glycerol concentrations in control for GSH process were also about 38% lower than that in the case of conventional dry grind process (0.93 vs. 1.51% w/v), which leads to higher ethanol production. Glycerol concentrations with germ water addition were 5% lower than that of control in GSH process (0.88 vs. 0.93% w/v). Similar to the observations in case of conventional process, the addition of protease enzymes resulted in complete fermentation; however, initial ethanol production rate was lower than that of with germ water addition (Fig. 6; Table 4). Ethanol yields of 0.52 and 0.53 L/kg dry grits were similar for germ water and protease addition but higher than that of control (0.48 L/kg dry grits).

**Table 4:** Ethanol yields and conversion efficiencies for all treatments in GSH process

Treatments	Final average ethanol concentration (%)	Average conversion efficiency (%)	Average fermentation rates (% v/v/h)
Control	15.10 c	76.75 c	0.245 e
Protease	16.63 a	84.58 a	0.378 d
Germ soak water 2 h 30 °C	15.47 b	76.60 c	0.431 c
Germ soak water 12 h 30 °C-PS	16.35 a	82.78 b	0.490 b
Germ soak water 12 h 30 °C-FS	16.63 a	84.09 ab	0.528 a
B-vitamins	16.43 a	83.91 ab	0.532 a
B-vitamins + germ soak water^a^	16.53 a	83.23 b	0.525 a

Means followed by the same letter in one column are statistically not different (at *P* < 0.05)

*PS* partial slurry, *FS* full slurry

^a^Partial slurry of 12 h 30 °C germ soak water

#### Effect of water amount

**Fig. 8:**
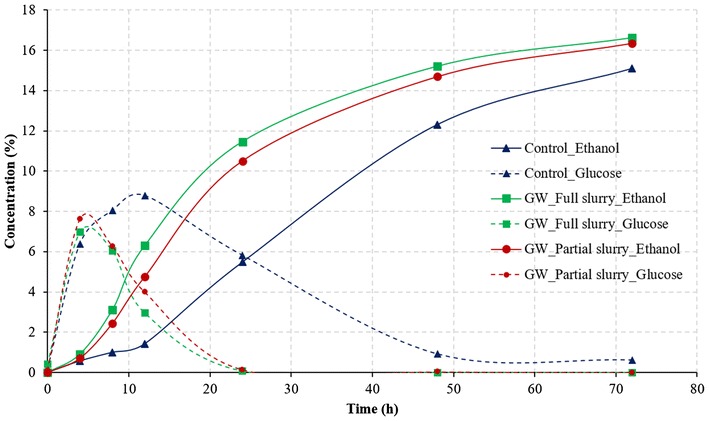
Effect of the amount of germ soak water on fermentation profile of corn grits in GSH process

Similar to the case of conventional process, the amount of germ soak water did not have a significant effect on the final ethanol concentration or conversion efficiency during GSHE process. However, the rate of ethanol production with full germ soak water was higher than the partial germ soak water (Fig. 8). This indicates that the micronutrients needed by the yeast were sufficient from the partial filtrate to obtain the maximum ethanol concentration at the end but higher nutrients in the full slurry lead the yeast to produce more ethanol at the beginning of the fermentation. There was complete fermentation at the end of 72 h as there was no residual glucose left.

#### Effect of B-vitamins

**Fig. 9:**
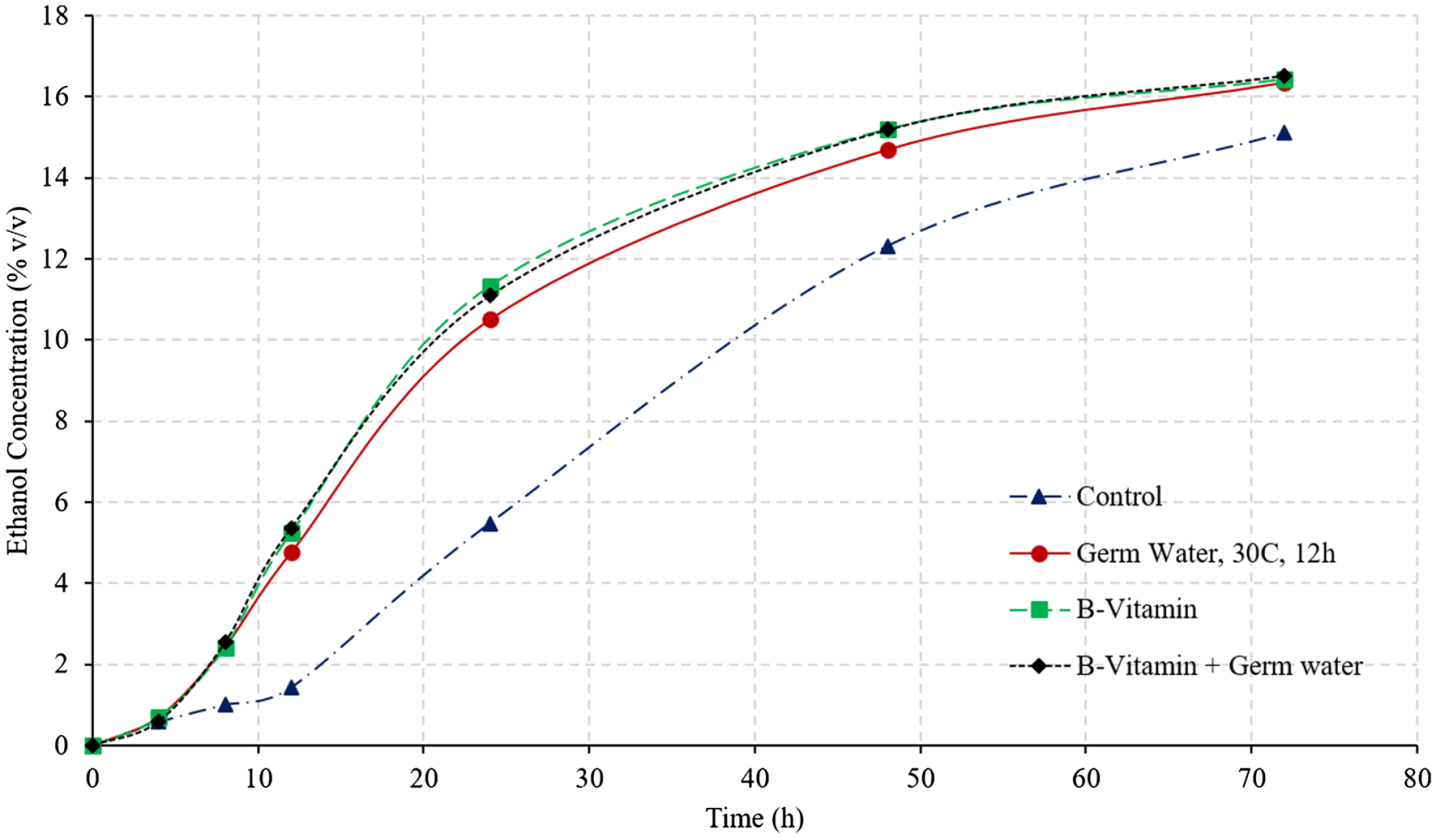
Ethanol concentrations during fermentation of corn grits in GSH process for control and treatments with germ water and B-vitamins supplementation

**Fig. 10:**
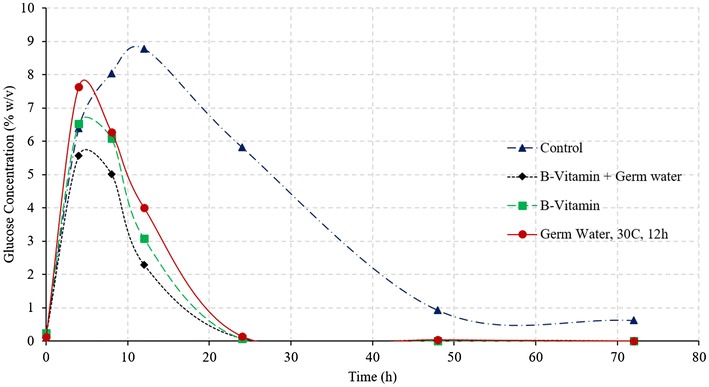
Glucose profile during fermentation of corn grits in GSH process for control and treatments with germ water and B-vitamins supplementation

The effect of external nutrients (vitamins B12 and B-vitamin complex) is shown in Figs. 9 and 10. The final ethanol concentration at the end of 72 h was statistically similar for germ water alone, B-vitamins alone, and both germ water and B-vitamins (Table 4). The rate of fermentation, however, was higher for added B-vitamin treatment (B-vitamin alone and B-vitamin with germ soak water). After the first 24 h, glucose was not detected in the fermentation slurry, indicating the conversion of glucose to ethanol by yeast was at the same rate of its formation by GSH enzymes. In the treatment of B-vitamin with germ soak water, the buildup of glucose is seen to be minimum, which suggests high conversion efficiency of glucose to ethanol in yeast. As it has been mentioned before, B-vitamins are essential for the yeast metabolism and aid in better functioning and increasing its stress tolerance (Branduardi et al. 2007; Laser 1941; Zhang et al. 2016). Final ethanol concentration of 16.5% was similar for all three treatments (germ water, B-vitamins, and combined germ water with B-vitamins). Starch-to-ethanol conversion efficiency was higher than 80% for all cases.

### Composition of germ

**Fig. 11:**
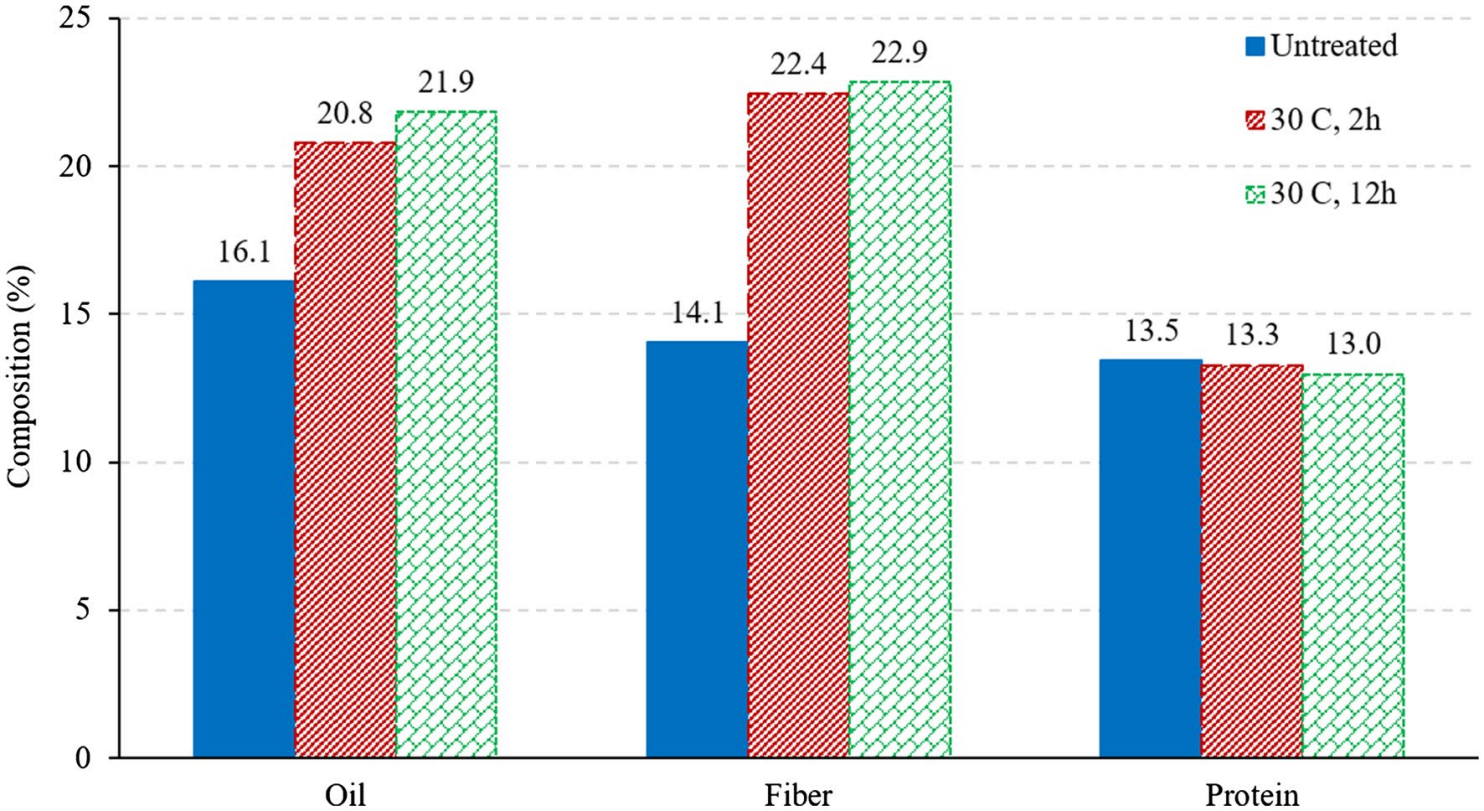
Composition analysis of remaining germ after water removal

Removal of soluble protein and micronutrients would potentially increase the oil content in germ and improves its economic value. Raw germ and germ obtained after soaking were analyzed for protein, oil, and fiber content. It can be observed from Fig. 11 that the oil concentrations in the germ increased by 29 and 36% for shorter and longer soaking conditions in comparison to that of raw germ. Since the market price of germ increases with its oil content (Johnston et al. 2005), the oil increase after soaking provides a huge advantage and makes this approach (germ soak water to improve fermentation) even more attractive. Compared to that of untreated germ, the protein concentrations of treated germ were about 1.5 and 3.7% lower for soaking conditions 30 °C, 2 h and 30 °C, 12 h, respectively. This indicates that during treatments, soluble micronutrients and proteins leached out in water, which when added to the fermentation broth, were available for the yeast to uptake and increased the fermentation efficiency.

## Conclusions

This study investigated and optimized the use of nutrient-rich water from corn germ soaking to improve fermentation of corn grits in comparison to through the use of protease enzymes or B-vitamin additions. Optimum soaking time and amount of germ water required was determined corresponding to maximum ethanol yield in conventional dry grind and granular starch hydrolysis process. The addition of germ water from soaking conditions of 30 °C for 12 h resulted in complete fermentation for both conventional and GSH processes, compared to significant residual sugars for control. Final ethanol yields were 29 and 8% higher than that of control in case of conventional and GSH process, respectively. GHS enzymes have previously reported to work better than conventional dry grind enzymes. However, the addition of germ water resulted in similar fermentation performance both GSHE and conventional enzymes. Initial ethanol production rates for samples supplemented with germ soak water were higher than that of samples supplemented with protease and similar to that from supplementation of B-vitamins for both processes. Due to leaching of micronutrients and soluble proteins, soaking process improved the oil concentrations in the germ, which would enhance its economic value. Overall, the use of germ water from optimum soaking conditions can potentially eliminate the need for protease enzymes or expensive nutrients addition for efficient fermentation, and provide other advantages of higher oil concentrations in germ, and potential of acid use reduction in the process.
